# 

**Published:** 1916-11

**Authors:** 


					﻿PUBLISHED MONTHLY.	ATLANTA	SUBSCRIPTION $1.00
JOURNAL-RE®!^?
OF MEDICINE^™5. '
(Atlanta Medical and Surgical Journal, Established 1855.	) pa j
bird ipar
and Southern Medical Record, Established 1870.	} VJI U I Lu I
Vol. LXIII Atlanta, Ga., November, 1916	No. 8
				

